# *In vivo* bioimaging with tissue-specific transcription factor activated luciferase reporters

**DOI:** 10.1038/srep11842

**Published:** 2015-07-03

**Authors:** Suzanne M. K. Buckley, Juliette M. K. M. Delhove, Dany P. Perocheau, Rajvinder Karda, Ahad A. Rahim, Steven J. Howe, Natalie J. Ward, Mark A. Birrell, Maria G. Belvisi, Patrick Arbuthnot, Mark R. Johnson, Simon N. Waddington, Tristan R. McKay

**Affiliations:** 1 Gene Transfer Technology Group, Institute for Women’s Health, University College London, 86–96 Chenies Mews, London WC1E 6HX, UK; 2 Stem Cell Group, Cardiovascular & Cell Sciences Research Institute, St. George’s University of London, Cranmer Terrace, London SW17 0RE, UK; 3 Department of Pharmacology, School of Pharmacy, University College London, 29–39 Brunswick Square, London WC1N 1AX, UK; 4 Wolfson Institute for Gene Therapy, Molecular and Cellular Immunology, Institute of Child Health, University College London, London WC1N 1EH, UK; 5 Faculty of Medicine, National Heart & Lung Institute, Imperial College, London, UK; 6 Wits/SAMRC Antiviral Gene Therapy Research Unit, Faculty of Health Sciences, University of the Witwatersrand, Johannesburg, South Africa; 7 Faculty of Medicine, Department of Surgery & Cancer, Imperial College, London, UK

## Abstract

The application of transcription factor activated luciferase reporter cassettes *in vitro* is widespread but potential for *in vivo* application has not yet been realized. Bioluminescence imaging enables non-invasive tracking of gene expression in transfected tissues of living rodents. However the mature immune response limits luciferase expression when delivered in adulthood. We present a novel approach of tissue-targeted delivery of transcription factor activated luciferase reporter lentiviruses to neonatal rodents as an alternative to the existing technology of generating germline transgenic light producing rodents. At this age, neonates acquire immune tolerance to the conditionally responsive luciferase reporter. This simple and transferrable procedure permits surrogate quantitation of transcription factor activity over the lifetime of the animal. We show principal efficacy by temporally quantifying NFκB activity in the brain, liver and lungs of somatotransgenic reporter mice subjected to lipopolysaccharide (LPS)-induced inflammation. This response is ablated in *Tlr4*^−/−^ mice or when co-administered with the anti-inflammatory glucocorticoid analogue dexamethasone. Furthermore, we show the malleability of this technology by quantifying NFκB-mediated luciferase expression in outbred rats. Finally, we use somatotransgenic bioimaging to longitudinally quantify LPS- and ActivinA-induced upregulation of liver specific glucocorticoid receptor and Smad2/3 reporter constructs in somatotransgenic mice, respectively.

Luciferase enzymes catalyze their substrates in a reaction that produces light; a reliable surrogate for relative protein expression. The application of luciferase as a reporter gene has been widely exploited in the study of promoter activity *in vitro*^1^ but its use *in vivo* has largely been restricted to evaluating gene delivery and marking engrafted cells^2^. The use of transcription factor (TF) activated reporter constructs whereby serial TF binding consensus sequences are engineered upstream of a minimal promoter driving luciferase is established. Such approaches have been used to define and quantify nuclear translocation and DNA binding of TF complexes to conditionally activate or repress gene expression. This approach has resulted in the definition of minimal DNA binding consensus sequences for the majority of characterized TFs. Such cellular responses to disease agonists are well studied in monolayer cultures *in vitro* but temporal tissue-specific studies hold far greater relevance to the understanding and treatment of disease. To interrogate disease progression at the molecular level it is necessary to study reliable biomarker activity in living animals over time but biomarkers are most often limited to blood and urine sampling.

The emergence of bioluminescence imaging (BLI) has enabled the use of promoter activated surrogate luciferase transgene activity as a biomarker in living small animals^2^,^3^,^4^. Photonic emissions from luciferase activity in the visceral organs of rats or mice can now be simply and accurately detected using comparatively inexpensive charge-coupled device (CCD) cameras. There has been some success in utilizing germline light producing transgenic (LPT) mice where luciferase is engineered downstream of endogenous gene promoters or a TF-activated construct inserted into a genomic “safe harbor” site. The advantage of this technology is that every cell contains a copy of the genetic biosensor therefore an accurate whole-body representation of the activity of any promoter/enhancer cassette is provided. However, this clonal ubiquity severely impedes bioluminescent monitoring of transcriptional activity in individual organs or tissues since the signal is difficult to resolve from “background noise” of adjacent tissues. Moreover, these transgenic strains are often developed and maintained on a limited number of genetic backgrounds, and therefore backcrossing is frequently necessary leading to increased costs and significant time delays.

Gene transfer of such TF activated reporters to adult rodents has been shown to result in loss of transduced cells through cellular immune responses^5^. Some success has been achieved by employment of immune-deficient mice^1^ however this complicates the study of TF activity in established transgenic or biological disease models where immune competency is required. We have discovered that lentivirus vector administration to neonatal rodents, before full maturation of the immune system, leads to immune tolerance to luciferase and permits lifelong transgene expression^6^. In addition, selection of the appropriate injection route and lentivirus pseudotype confers organ and tissue selectivity. To enable tissue-specific measurement of the activity of virtually any TF we have generated a parental lentivirus construct to shuttle serial TF consensus binding sequences upstream of an *in vivo* optimized firefly luciferase (FLuc)/eGFP expression cassette. High-titer lentivirus is targeted to tissues using a combination of route administration and viral pseudotyping in newborn neonates. This achieves substantially greater organ spread than in adults and induces immune tolerance to the transgenic proteins, thus permitting lifelong conditional expression. Experimentation on these somatotransgenic animals is quick, taking less than 2 months from selection of TF to data acquisition, is cost effective in comparison to LPT production/colony maintenance and crucially is tissue-specific.

We have developed this methodology as a platform to study TF activity during disease pathogenesis or for pre-clinical evaluations of drug efficacy. We present data showing the utility of this approach by modeling NFκB activity in living mice and rats in response to pro-inflammatory challenge. Early inflammatory responses are mediated through NFκB signaling in most tissues. Bacterial infection or pro-inflammatory cytokines such as TNF-α activate the IκB kinase (IKK) which phosphorylates the NFκB repressor IκB, targeting it for ubiquitination and proteasomal degradation^7^. Free NFκB dimers translocate to the nucleus where the p50 protein directly associates with the 5′-GGGACTTTC-3′ consensus DNA sequence in the promoters of NFκB target genes initiating an inflammatory response^8^. We generated a FLuc/eGFP reporter lentivirus under the conditional control of 8x serial NFκB binding sequences. We demonstrate the utility of resultant tissue specific NFκB reporter rodents as a drug evaluation platform for the assessment of anti-inflammatory substances. Similarly, we show repression of NFκB activity but activation of a similarly designed glucocorticoid receptor response element (GRRE) reporter by the corticosteroid analogue dexamethasone. Finally, to show activation of a further commonly investigated signaling pathway we show that liver specific TGF-β activated Smad2/3 reporter mice are responsive to a single intraperitoneal dose of the TGF-β superfamily ligand, Activin A.

## Results

### Generation of a library of lentivirus vectors containing transcription factor activated reporters

**Figure 1:**
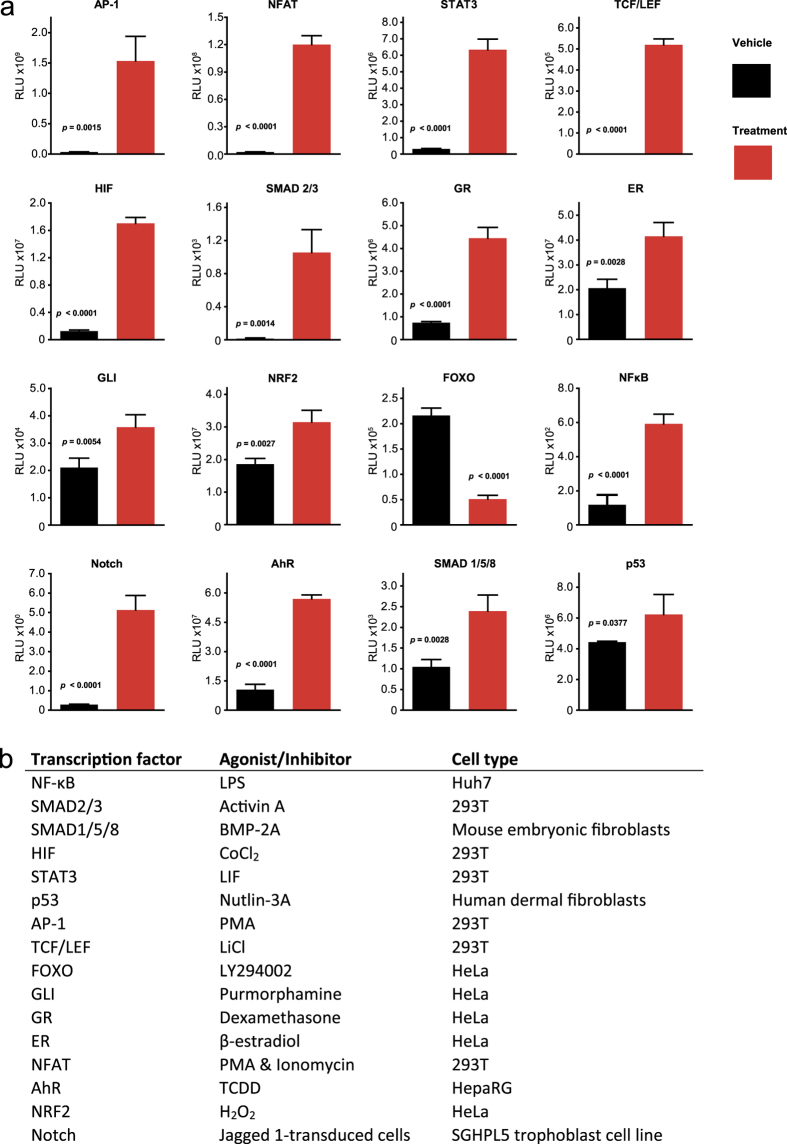
*In vitro* validation of a library of transcription factor activated lentiviral reporter vectors. Cells were transduced with the VSV-G pseudotyped lentiviral vectors at an MOI of 10 then incubated with the relevant agonists/inhibitors for the appropriate duration (Supplementary Table 2). (**a**) Each treatment induced a significant effect in cells containing the respective transcription factor response element between 24 and 72 h post-treatment (n = 3–6, mean +/− SEM, comparison by Student’s t-test). (**b**) A list of transcription factors, agonist/inhibitors and cell types.

First we sought to generate a library of widely applicable TF activated reporter lentiviral constructs encompassing many of the commonly investigated signaling pathways in biomedical research. We constructed a parental lentiviral vector with a Gateway® acceptor site upstream of a minimal promoter driving a codon optimized, heat stabilized FLuc/eGFP bicistronic expression cassette (Supplementary Fig. 1). By *de novo* synthesizing serial repeats of known TF consensus sequences and rapid Gateway® cloning into the parental cassette we have designed and generated a preliminary library of 16 TF activated reporter lentiviral vectors. We transduced cell lines at a multiplicity of infection of 10 with these vectors and stimulated the reporters with appropriate pathway agonists/antagonists. All reporters showed significant specific responses which varied in time and amplitude depending on the reporter, cell line and agonist/antagonist (Fig. 1a). This was expected as biological responses will alter in length and magnitude as well as varying between cell types.

### Administration of lentivirus containing NFκB reporter to rodents to examine feasibility of organ targeting, durability of expression, and LPS dose-responsiveness

**Figure 2:**
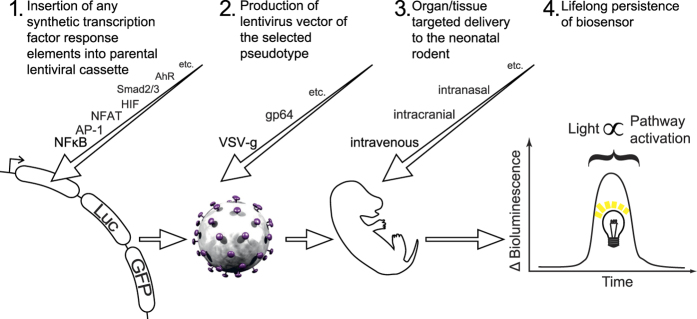
Schematic representation of the Somatotransgenic BLI methodology. Serial transcription factor binding sequences are *de novo* synthesized and inserted upstream of a minimal promoter driving a FLAG-tagged *firefly luciferase*-2A-eGFP lentiviral expression cassette. High titer lentiviral preps are generated with tissue tropic pseudotyped envelope proteins. Lentivirus is administered to neonatal rodents by various routes and the degree of bioluminescence observed is proportional to the activation of the pathway controlled by the associated transcription factor.

Initial proof-of-concept *in vivo* experiments were designed to assess whether long-term biosensing of transcription factor activity could be achieved in an organ or tissue-specific manner following neonatal lentivirus vector administration (Fig. 2). To assess optimal dose, NFκB reporter lentiviral vectors pseudotyped with the wide tropism VSV-G envelope were injected intravenously into newborn (P0/1) outbred mice. We consistently used doses of ≈2 × 10^7^ vp/mouse in all subsequent experiments, regardless of administration route. Intravenous injection of VSV-G pseudotyped vector in neonates results in targeted transduction of the liver as determined by *ex vivo* tissue luminometry (Supplementary Fig. 2). As predicted we observed a dose response in FLuc expression to LPS optimal at 1,200 ng/kg (Supplementary Fig. 3). We have endeavored to construct a method of data analysis which accommodates for sources of error and minimizes subjective interpretation. If data is presented as total bioluminescence (Supplementary Figure 4) there is variation in initial, baseline bioluminescence, which may be a consequence of genetic diversity (these are outbred mice) and differences in vector delivery and infection efficiency. Therefore we have calculated and presented all data as fold increase over baseline – Δ bioluminescence. To ensure this baseline is representative of true basal bioluminescence, we have derived the median of three consecutive images, taken on consecutive days where possible. Median, unlike mean, is a robust measure of central tendency and therefore would be unaffected by an outlier in the baseline triplet. Supplementary Figure 4 reveals that total bioluminescence fell around fourfold from birth to around 40 days of life, then fell around 50% again over the ensuing 200 days. However, a fall in overall signal does not appear to correlate with diminished sensitivity, and a second dose of LPS induced a strong NFκB response which was not significantly different from the response to the first LPS dose.

### Liver and brain targeting

**Figure 3:**
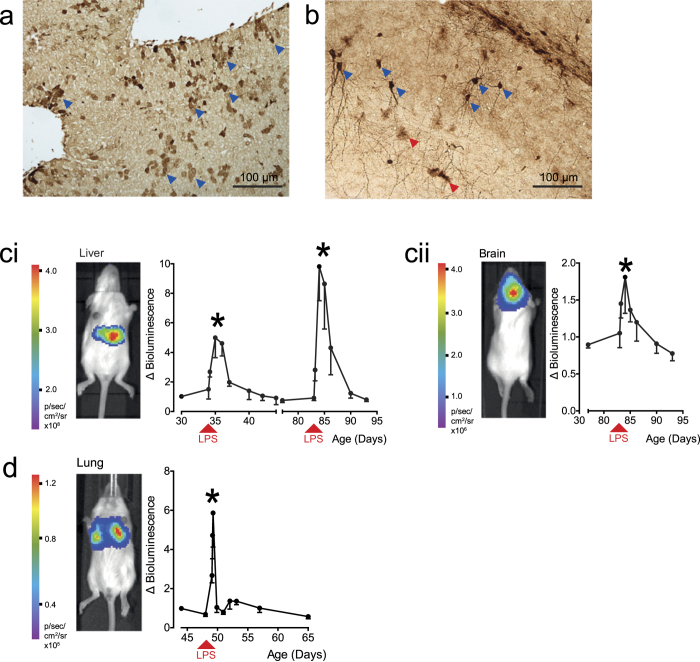
Lipopolysaccharide (LPS)-mediated induction of NFκB-activated reporter cassette in targeted organs of reporter mice. Preparations of NFκB reporter lentiviral vector pseudotyped with VSV-G were administered to newborn outbred mice by intravenous (n = 9) or intracranial (n = 9) injection to target liver and brain, respectively. After establishing a baseline at 34 days, reporter mice were challenged with LPS and BLI measured continually thereafter. After 24 h mice receiving intravenous and intracranial injections were sacrificed and GFP immunohistochemistry performed on (**a**) liver and (**b**) brain sections, respectively. Liver staining was found predominantly in hepatocytes (**a**, blue arrows); Brain GFP expression was seen mainly in cells of neuronal (**b**, blue arrows) and astrocyte (**b**, red arrows) morphology. Photon emission was restricted to **(ci, image)** liver and **(cii, image)** brain, respectively. Both (**ci, graph**) liver and (**cii, graph**) brain showed acute early responses to LPS stimulation. Baseline BLI was re-established in liver specific animals and were re-stimulated at 83 days and imaged over the same timeframe. The NFκB reporter lentiviral vector was pseudotyped with gp64 envelope for (**d**) nasal administration to the lung airway epithelium of neonatal mice. Adult mice were challenged with LPS at 48 days (n = 4) and BLI quantified continually thereafter. Lung targeted somatotransgenic animals showed an acute response to LPS (*P < 0.01, repeated measures ANOVA). Images are presented as captured at the time of peak bioluminescence for each experiment.

For evaluation of LPS-induced inflammation, the NFκB reporter lentiviral vector was administered by intravenous or intracranial injection to target the liver and brain, respectively. Immunohistochemical staining for GFP in livers collected 24 hours after LPS stimulation revealed predominant expression in hepatocytes (Fig. 3a, blue arrows) although occasional staining of biliary epithelium and Kupffer cells was evident. Intracranial administration showed widespread transduction with GFP expression in cells predominantly displaying neuronal and occasional astrocyte morphology (Fig 3b, blue and red arrows, respectively). Bioluminescence persisted in all tissues targeted for at least 7 months (data not shown). To determine the effect on reporter gene activity in individual animals, each data point was measured as a fold-change over the baseline. A single challenge with LPS induced marked up-regulation of luciferase activity in liver and brain within 24 hours, which returned to pre-challenge levels within 7 days (Fig. 3ci & ii; n = 8 per group, P < 0.01). Furthermore, we show that animals can be re-used in repeat experiments by re-administration of LPS 47 days after the first dose in liver specific NFκB somatotransgenic mice. A second acute peak in luciferase activity was observed that was significantly higher than the initial response (P<0.01). Bioluminescence signal was detected in all treated mice for the duration of the experiment.

### Lung targeting

Since vectors pseudotyped with the VSV-G protein transduce pulmonary airway epithelium poorly, we generated vectors pseudotyped with the baculovirus envelope glycoprotein, gp64, which mediates efficient epithelial transduction in neonatal lungs^9^. Lung-specific NFκB reporter mice were generated by intranasal administration of the vector to newborn animals. LPS was then administered by nebulization to the adult mice. The minimal efficacious dose was determined by counting total leukocytes, neutrophils and eosinophils in bronchoalveolar lavage fluid (Supplementary Fig. 5). Optimized LPS nebulization induced a peak in pulmonary luciferase expression at 24 h, which returned to baseline within 48 h (Fig. 3d; n = 4, P < 0.01).

### Confirmation of biosensor specificity

**Figure 4:**
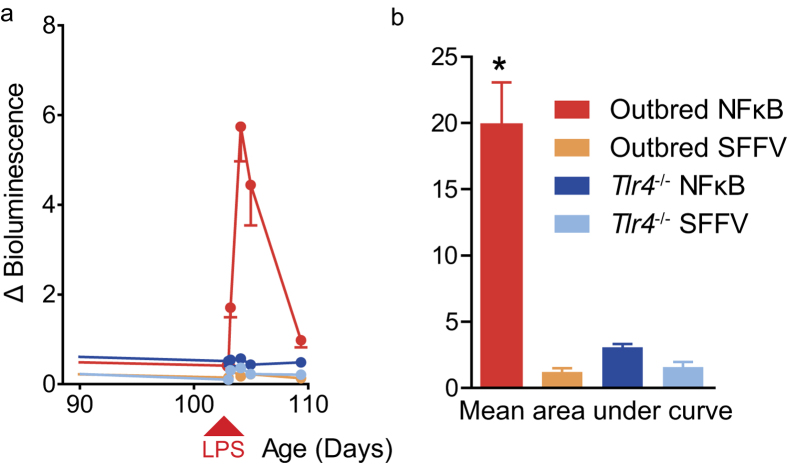
NFκB reporter activation by LPS is TLR4 dependent in liver targeted somatotransgenic mice. Preparations of NFκB reporter lentiviral vector pseudotyped with VSV-G were injected intravenously into newborn outbred mice to achieve liver-targeted expression (**a**) Liver-specific NFκB pathway activity was determined by LPS challenge in outbred NFκB (n=21) and constitutively active SFFV reporter (n = 7) mice versus *Tlr4*^−/−^ NFκB (n = 9) and SFFV (n = 7) reporter mice. (**b**) Only wild-type NFκB reporter mice showed significant increase in luciferase activity (*P<0.01, repeated measures ANOVA).

To confirm specificity of the biosensor response to NFκB activation, we compared the effects of LPS on outbred and *Tlr4*^*−/−*^ mice. Animals were injected intravenously with vector encoding luciferase/eGFP under control of either the constitutively active promoter derived from spleen focus-forming virus (SFFV), or the NFκB-responsive transcription element. LPS induced a statistically significant peak of bioluminescence in outbred NFκB reporter mice (Fig. 4a,b; n = 7–21; one-way ANOVA *P *< 0.01), compared to no response in *Tlr4*^*−/−*^ mice. This demonstrates that, as with our *in vitro* validation, the NFκB biosensor was specific for TLR4-dependent induction of NFκB by LPS. For experiments like this, with two or more treatment groups possessing the same biosensor, we derived an area under curve for each group. We felt this was more representative of total transcription factor activity over the duration of the experiment and provided a more objective method of analysis for further experiments. Therefore we have presented a kinetic profile of bioluminescence as well as area under curve. Overall, absence of an effect in either strain of mice treated with the vector encoding the SFFV promoter indicates that the NFκB binding sequences alone, not the lentiviral vector backbone, conferred sensitivity to LPS-induced activation of transcription by NFκB.

**Figure 5:**
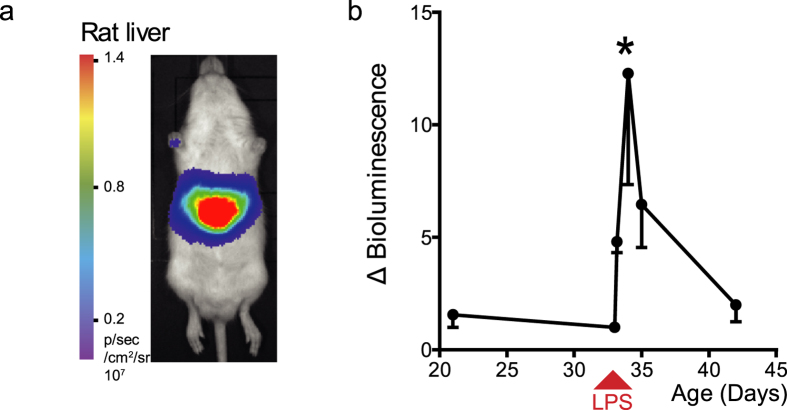
Lipopolysaccharide (LPS)-mediated induction of liver specific NFκB-reporter rats. Liver biosensing rats were generated by injection of VSV-G-pseudotyped NFκB reporter lentivirus (titer 5 × 10^9^ vp/ml) intravenously into the superficial temporal vein of newborn Sprague-Dawley rats (n = 6). 50 μl LPS (125 μg/kg) was administered by intra-peritoneal injection at 33 days and BLI carried out at 12, 24, 48 and 168 h. (**a**) As in mice, expression was localized to the liver and (**b**) there was a significant acute early response in luciferase expression, peaking 12 hours after LPS administration (*P < 0.01, repeated measures ANOVA).

To evaluate the transferability of the somatotransgenic approach to alternative rodent species, we targeted the livers of Sprague-Dawley rats using the same NFκB biosensing lentiviral vector (Fig. 5a,b; n = 7). Challenge with LPS induced a significant increase in BLI, which was similar to that seen in mice (P < 0.01).

### Using light producing somatic transgenic mice for *in vivo* analysis of drug efficacy

**Figure 6:**
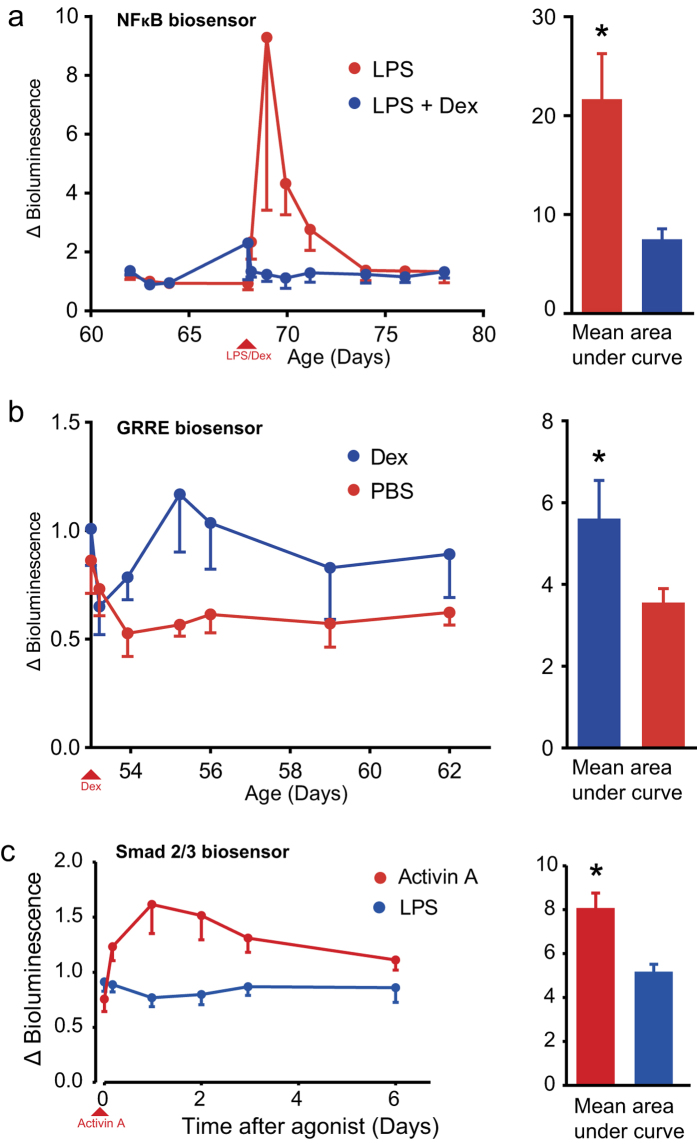
Reporter mice as an *in vivo* drug evaluation platform using continued BLI imaging. For all three experiments, outbred mice received neonatal intravenous injection of ≈2 × 10^7^ vp/mouse of VSV-G pseudotyped vector to achieve liver-targeted expression of different reporters. (**a**) NFκB reporter mice were treated with either dexamethasone (113 μg/mouse, n = 8) or vehicle (n = 8) at −2 h and concomitantly with LPS challenge. There was a significant suppression of BLI in the dexamethasone treatment group (*P = 0.047). (**b**) GRRE reporter mice were treated with either dexamethasone (450 μg/mouse, n = 8) or vehicle (n = 8) and subjected to BLI over 14 days. There was a significant upregulation of BLI in the dexamethasone treatment group (*P < 0.05). (**c**) Smad2/3 reporter mice received an i.p. injection of Activin A. Control mice received i.p. LPS. An acute Smad2/3-specific BLI response was observed after Activin A but not LPS (n = 13 each group; *P *< 0.01, Student’s t-test).

Liver-specific NFκB reporter mice were used to assess inhibition of pro-inflammatory transcriptional activation by an anti-inflammatory drug. The glucocorticoid analogue dexamethasone is known to inhibit NFκB activity through multiple direct and indirect mechanisms^10^. Mice received LPS with or without dexamethasone and LPS induced an NFκB-mediated up-regulation in luciferase expression, which was completely ablated by dexamethasone (Fig. 6a; n = 8 per group; one way ANOVA *P *< 0.05). This validation of a known anti-inflammatory drug proves that this protocol may be employed for drug evaluation using real time analysis *in vivo*. As well as inhibiting NFκB-mediated inflammation, dexamethasone will also upregulate the glucocorticoid receptor (GR) response resulting in the activation of gene promoters containing GRRE^11^. We generated liver-targeted GR reporter mice and subjected them to intra-peritoneal injection of dexamethasone. In this case, we observed a significant upregulation of FLuc expression for two days following dexamethasone administration (Fig. 6b; n = 8 & 7; ANOVA *P *< 0.05). Together these data show that we are able to use multiple transcription factor-activated reporters to interrogate dual activity of a single drug. Finally, we sought to show direct downstream transcription factor activity after applying a cytokine *in vivo*. We generated liver-targeted Smad2/3 reporter somatotransgenic mice as a biosensor for the TGF-β family member Activin A. Smad2/3 somatotransgenic mice received an intraperitoneal injection of Activin A whereas control mice received LPS as a non-activating control. An acute BLI response after Activin A but not LPS demonstrated Smad2/3-specific biological sensor activity and no cross-activation (Fig. 6c; n = 13 per group; one-way ANOVA *P *< 0.01).

In conclusion, these experiments describe generation of a library of transcription factor-activated reporters, using a technology which can be used easily and inexpensively to vectorize any desirable response element. We demonstrate that neonatal administration of appropriately pseudotyped lentivirus vector by the appropriate route can target liver, brain and lung. This is achieved in outbred mice, knockout mice, and rats. Using NFκB as an example, we observe that the biosensor acts in a dose-dependent manner to the LPS agonist, and that it is specific to the toll-like receptor 4 signaling pathway. Finally, we have been able to show utility, in the liver, in assessing drug efficacy, using dexamethasone, and have shown that an additional biosensor, Smad2/3, responds to the ligand Activin A.

## Discussion

Luciferase assay is established as the predominant modality of transcriptional activity studies *in vitro* and now luciferase BLI is becoming more commonplace in living animals. However, continual BLI in rodents has been employed largely to quantify bacterial colonization^12^ and engraftment of cancer cells^13^ and stem cells^14^. Most recently, germline LPT mice have been generated by inserting the luciferase gene downstream of an endogenous gene promoter to act as a quantifiable surrogate for gene expression in living animals. Since Contag *et al.*^15^ first described germline LPT mice, the diverse utility has been evidenced in studies of development^16^, normal physiology^3^ and disease pathogenesis^17^ including oncogenesis and metastasis^2^,^4^. A further technological iteration has been the genomic insertion of conditional expression cassettes where luciferase is activated by serial transcription factor binding sequences driving a minimal promoter^18^. In this context, TF activity can thus be discriminated from promoter activity.

To date there have been over twenty LPT mice described in the literature, ten of which contain transcription factor activated synthetic promoters. Since they possess an integrated luciferase sensor cassette, production of mice for experiments requires nothing more than breeding, rather than the technique described herein, of neonatal vector injection. However, the initial generation of transgenics is extremely time consuming and the lines are costly to produce and maintain; it is estimated that from transfection of ES cells to the first litter of homozygous mice takes approximately one year at a cost of approximately $12,000^19^. Targeted knock-in transgenics may also be compromised by off-target activity^20^,^21^. For example, Ciana *et al.* showed that an estrogen receptor-responsive light producing transgenic was subject to both variation in tissue activity and agonist activation in F_1_ progeny^22^. This variation could be caused by positional effects with the inserted transgene, also reported by Zinn *et al.*^23^. Avoiding this effect would require extensive analysis of F_1_ progeny and therefore adds to the time and cost of developing the animal model.

We have developed an alternative approach that enables rapid generation of mice with tissue-restricted bioluminescent reporter activity. The combination of lentiviral pseudotyping and neonatal administration to target specific tissues allows for the generation of a large variety of somatic transgenic rodents. Production is rapid and cost effective compared with generation of germline transgenic rodents. There are several previous studies describing production of somatic, transgenic, light emitting mice by adult gene delivery. For example hydrodynamic plasmid injection has been used to monitor activity of the hepcidin promoter^24^; a separate study described intramyocardial injection of plasmid containing a heart-specific promoter^25^. Intra-tumoral injection of adenovirus vector was used to measure alpha-fetoprotein-promoter/enhancer activity^26^. Adenovirus vector was injected into distinct CNS nuclei to measure NFκB activity^27^. However we^28^ and others^29^ have demonstrated that systemic vector delivery can provoke an immune response which can eliminate transduced cells whereas neonatal delivery can induce CD4^+^ CD25^+^ T cell-mediated tolerance to the transgenic protein^30^. Specific administration of lentivirus vector to the perinatal rodent results in lifelong transgene expression^31^,^32^. Unlike germline transgenic rodents which may occasionally suffer from positional effects exerted by regulatory elements proximal to the integration site, retroviral vectors integrate in a semi-random pattern therefore overall expression is unlikely to be influenced by any single local enhancer or repressor.

We chose to concentrate our evaluation of this technology on an NFκB reporter construct for two reasons: Firstly, NFκB is a mediator of the early inflammatory response and hence its activity and modulation is important in the pathogenesis of many disease states and is a target for anti-inflammatory drugs. Secondly, a light-emitting germline NFκB transgenic mouse model is established^33^,^34^,^35^ but not widely used because of problems associated with substantial multi-tissue background signal. NFκB activity plays multiple roles in proliferation, apoptosis and stem cell differentiation^8^ within normal tissue homeostasis. Consequently, off-target bioluminescence activity renders BLI of visceral tissues practically impossible in germline NFκB transgenic models.

We generated liver, brain and lung targeted NFκB reporter mice and showed repeated acute responses to LPS. In the liver we observed a greater response to the second LPS administration compared with the first. This contradicts many studies showing that repeated LPS dosing induces tolerance. However, many of these studies have used readouts downstream of the primary signaling pathways (e.g. leukocyte infiltration, nitric oxide production) whereas here, we have been uniquely able to quantitate NFκB activation. Other rodent models are used in biomedical research and we have shown that this technology can be also be applied across any species that is compatible with *in vivo* bioimaging by producing LPS responsive liver specific NFκB somatotransgenic rats. A further substantial advantage of somatotransgenic technology is that it can be conveniently layered upon existing transgenic mouse strains. We took advantage of this to prove that the NFκB- reporter was stimulated directly via LPS/TLR4-mediated NFκB signaling. Ultrapure LPS-induced BLI responses were completely ablated in *Tlr4*^*−/−*^ mice compared to wild-type controls or a promoter not influenced by NFκB (SFFV). This proves at a cellular level that NFκB-mediated expression of luciferase is regulated through TLR4. Although this data is proof-of-principle it shows that somatotransgenic BLI can potentially be used to verify the downstream function of any protein in knockout mice where there is downstream modulation of transcription factor activity.

A widely applicable utility of this technology is in drug efficacy studies. We used liver targeted NFκB reporter mice to show that intraperitoneal administration of the anti-inflammatory glucocorticoid analogue dexamethasone, prior to giving LPS, completely ablated the temporal NFκB BLI response. This shows that such an *in vivo* platform could be used in the early stage evaluation of potentially therapeutic compounds. This is useful not only in the study of inflammation but in analysis of any drug that is designed against a protein target whose activation or repression results in modulation of transcription factor activity. Finally, we sought to show that a defined TF activated reporter was upregulated directly by a cytokine *in vivo*. We achieved this by generating a Smad2/3 activated reporter which is responsive to TGF-β family members. Liver specific Smad2/3 somatotransgenic mice showed a significant response to a single intraperitoneal dose of Activin A compared to no response to LPS as a non-activating control. This shows that *in vivo* TF-activated reporters are faithful to predictive biological agonists. In this study we have shown proof of principle for bioluminescence imaging of transcriptional activation in three organs – liver, lung and brain. However, the technology should be applicable to any tissue or organ which can be transduced by lentivirus vector. This, in turn, depends upon, and is restricted by, route of administration and the vector pseudotype.

The 3Rs principles are to reduce, refine or replace the use of animals in biomedical research, wherever possible. BLI using germline or somatotransgenic mice substantially reduces the number of animals necessary for longitudinal studies of gene expression compared to conventional endpoint analyses as well as increasing quality by maintaining continuity within experiments. In summary we have developed a new methodology for rapidly generating tissue-specific BLI rodent models for use in disease pathogenesis and drug efficacy studies. Somatotransgenic BLI can be applied to the study of a wide array of signaling networks. We have generated and *in vitro* validated an initial library of 16 lentiviral reporter cassettes suitable for interrogating transcription factor activity in proof of principle studies using NFκB, GR and Smad2/3 reporters. Using this new technology, kinetic profiles of signaling pathways can be obtained using smaller groups of animals, at a lower cost and in a shorter period of time.

## Methods

### Construction of lentiviral reporter vectors

Triple overlap-extension PCR was performed using Phusion high-fidelity polymerase (NEB) and primer sets to amplify 3xFLAG, FLuc, and 2A-eGFP. 2A is the Foot and Mouth Disease Virus 2A cleavage peptide sequence and serves as a polycistronic linker, as previously described^36^. The upstream primer (F1) was engineered to contain a unique *Bam*HI restriction site, while the downstream primer (R3) contained a unique *Mlu*I site. A single in-frame cistron was generated by overlap-extension secondary PCR using the three primary PCR products as template and 3xFLAG (F1) and 2A-eGFP (R3) primers for amplification. This produced the *Bam*HI-FLAG-Luc-2A-eGFP-*Mlu*I fragment which was sub-cloned into an intermediary plasmid using the *Bam*HI/*Mlu*I sites and then shuttled into our parental pLNT-Gateway-MCS vector using *Sal*I/*Mlu*I into *Xho*I/*Mlu*I to create pLNT-Gateway-JDG (Supplementary Fig. 1). To shuttle transcription factor activated promoters into the Gateway® recombination site we first cloned the adenoviral E1a minimal promoter sequence (MP) into the Gateway® shuttle vector pENTR-1A (Invitrogen) by hybridizing overlapping oligonucleotides MP (F & R) both containing terminal *Xho*I sites. This product was then ligated into the unique *Xho*I site of pENTR-1A and correctly oriented clones confirmed by sequencing. This plasmid (pENTR-MP) acted as an intermediary shuttle vector into which serial transcription factor binding elements (TFBE) were cloned (Table 1). TFBE were *de novo* synthesized by Aldevron (Fargo, ND, USA) and ligated into pENTR-MP using unique *Bam*HI/*Eco*RI sites. Sequence verified TFBE-MP sequences were recombined into pLNT-Gateway-JDG using Gateway® cloning technology (Invitrogen) as per manufacturer’s instructions.

### Lentivirus production and titering

293T viral producer cells were seeded overnight at 2 × 10^7^ cells per T175  cm^2^ flask and transfected using 50 μg pLNT-TFBE-JDG, 17.5 μg pMD.G2 (VSV-G envelope plasmid), and 32.5 μg pCMVΔR8.74 (gag-pol packaging plasmid) pre-complexed with 1 μl polyethylenimine (10 mM) (Sigma-Aldrich) in OptiMEM for 3 h. Transfection media was replaced with complete DMEM and viral supernatant collected at 48 and 72 hours, filter sterilized (0.22 μM), and concentrated by overnight low-g centrifugation. Pellets were resuspended in OptiMEM and stored at −80 °C. All viruses were titered using a p24 assay (Zeptometrix, Buffalo, NY, USA) as per manufacturer’s instructions.

### *In vitro* validation of reporter gene activity

293T, HeLa and NIH3T3 cells were cultured in DMEM supplemented with 10% fetal bovine serum, 1% penicillin/streptomycin (Sigma), 2 mM L-glutamine and 1x non-essential amino acids. HepaRG cells were cultured in William’s E media containing Glutamax and sodium pyruvate supplemented with 10% FBS, 1% Pen/Strep, 1x non-essential amino acids, 4 μg/ml human recombinant insulin zinc solution, and 50 μM hydrocortisone. All reagents were sourced from Life Technologies.

Lentiviral expression constructs were initially validated using an SFFV constitutive promoter driving expression of the firefly luciferase-eGFP bicistronic reporter. Efficient 2A cleavage was confirmed by western blot for FLAG (Sigma-Aldrich F7425; 1:1,000) and eGFP (AbCam Cat: Ab290; 1:10,000) using standard methodologies and Fluorescence Activated Cell Sorting using a FACS Calibur flow cytometer, data capture using Cell Quest and data analysis using FlowJo v7.6.4 software (data not shown).

For the validation of transcription factor activated gene expression *in vitro* (except Notch), we transduced cells with VSV-G pseudotyped lentivirus reporters at an MOI of 10 then applied small molecule or growth factor agonists or inhibitors as detailed in Fig. 1b and Supplementary Table 2. For analysis of the Notch reporter we transduced the trophoblast isolated cell line, SGHPL5, then mixed with HEK93T cells transduced with Jagged-1 expressing lentivirus or empty vector and co-cultured for 72 h prior to analysis.

Gene activation was quantified by luciferase luminometry on cell lysates as follows: 20 μl assay buffer (25 mM Tris phosphate pH 7.8, 1mM DTT, 1 mM EDTA, 1% Triton X-100, 8 mM MgCl_2_, 3 ml glycerol, 1.25 mM rATP, 0.5% BSA) was added to 20 μl cell extract. Luciferin (Gold Biotechnology, MO, USA) was added to a final concentration of 1.5 mM and luminescence measured using the POLARstar Omega microplate reader (BMG Labtech). Analysis performed using MARS data analysis software (BMG Labtech). Relative light units were normalized relative to total protein as determined by Bradford assay (BioRad) using manufacturer’s instructions.

### Animal procedures

Unless otherwise stated, outbred CD1 mice (Charles River) were time mated to produce neonatal animals. At postnatal day 1, neonates were briefly anesthetized (on ice) and lentivirus injected by the appropriate route: Intravenous (via the superficial temporal vein); 20 μl, Intracranial; 5 μl. Intranasal insufflation was achieved by restraining the neonate with mouth closed while 10 μl of vector was placed on the nostrils. Neonatal outbred Sprague-Dawley rats (Charles River) received 50 μl of intravenous lentivirus. Mice deficient in the toll-like receptor 4 (TLR4^−/−^) were obtained from the Swiss Immunological Mouse Repository (SwImMR) and outbred onto the CD1 background. All experiments were performed in accordance with relevant guidelines and regulations: Experiments were carried out under United Kingdom Home Office regulations and approved by the ethical review committees of Imperial College London and University College London.

### Bioluminescence imaging

Unless otherwise stated, animals were anesthetized with isoflurane (Abbott Laboratories), injected i.p. with firefly D-luciferin (15 mg/ml in PBS; Gold Biotechnology) and imaged 5 min later with a cooled charge-coupled device (CCD) camera (IVIS; PerkinElmer). Luciferin dose was 150 mg/kg; volume varied with age/size of animal, for example, adult mice were given 300 μl. Grey-scale photographs were acquired with a 24-cm field of view and then a bioluminescence image was obtained using a binning (resolution) factor of 4, a 1.2/f stop and open filter. Regions of interest (ROIs) were defined manually using a standard area for each organ under investigation. Signal intensities were calculated with Living Image software (Perkin Elmer) and expressed as photons per second per cm^2^ per steradian. Where possible, BLI was carried out in adult reporter rodents on three consecutive days to establish a robust median baseline; subsequent data points were expressed as fold-change over this internal standard for each individual animal.

### *In vivo* agonist activation

LPS was given i.p., at a dose of 30 μg/mouse or 18.75 μg/rat. For NFκB BLI (Fig. 5a), dexamethasone was given i.p. 2 hours before and concomitantly with LPS and luciferin, at a dose of 113 μg/mouse. For GRRE BLI (Fig. 5b) a dose of 450 μg/mouse dexamethasone was injected. Activin A was injected i.p. at a dose of 25 μg/mouse.

### Immunohistochemistry

Brain was processed as previously described^37^. Whole brain was fixed in 4% paraformaldehyde. The brains were cryoprotected in 30% sucrose in 50 mM TBS prior to cryosectioning at 40-μm sections using a Microm freezing microtome (Carl Zeiss, Welwyn Garden City, UK). eGFP expression was detected by immunohistochemical staining of the sections. Endogenous peroxidase activity in the sections was blocked by incubation in 1% H_2_O_2_ in TBS solution for 30 min. The sections were then rinsed three times in TBS before blocking of non-specific immunoglobulin binding by incubating sections for 30 min in a solution of 15% normal goat serum (NGS) in TBS-T (TBS solution containing 0.3% Triton X-100). Sections were then incubated overnight at 4 °C in rabbit anti-GFP (1:10 000; Abcam, Cambridge, UK) in TBS-T/ 10% NGS. The following day, the sections were rinsed in TBS and incubated for 2 h in goat ant-rabbit IgG diluted in TBS-T/10% NGS, rinsed again in TBS and incubated for 2 h in a 1:1000 solution of Vectastain avidin-biotin solution (ABC; Vector Labs, UK) in TBS prepared 30 min before use. Subsequently, sections were rinsed again and incubated in a freshly made and filtered 0.05% solution of DAB containing 0.01% H_2_O_2_ to visualize GFP immunoreactivity. The staining reaction was stopped by rinsing in ice-cold TBS. Stained sections were subsequently mounted onto chrome gelatine coated Superfrost-plus slides (VWR, Poole, UK) and left to dry overnight. Sections were then dehydrated in a series of rising concentrations of industrial methylated spirits, cleared in xylene for 20 min and coverslipped using DPX mounting medium (VWR). Liver tissue was fixed in 25% formalin overnight, transferred to 70% ethanol, and processed into paraffin. eGFP immunohistochemistry was performed on mounted, rather than free-floating sections.

### Statistical analysis

Statistical analysis on *in vitro* vector analysis data was performed using an unpaired Student’s one-tailed t-test. All data are expressed as mean values ± SEM, with each sample being measured at least in triplicate. For *in vivo* data, repeated measurements following agonist administration were analyzed with repeated measures analysis of variance with significance level of P < 0.01. Area under curve data for experiments with two groups were analyzed by Student’s one-tailed t-test. For areas under curve derived from two or more groups, one-way analysis of variance with Newman-Keuls post-hoc multiple comparison test was performed. For lung leukocyte analysis, one-way ANOVA with Dunn’s post-hoc test against vehicle control was performed.

## Additional Information

**How to cite this article**: Buckley, S. M. K. *et al.* *In vivo* bioimaging with tissue-specific transcription factor activated luciferase reporters. *Sci. Rep.* **5**, 11842; doi: 10.1038/srep11842 (2015).

## Supplementary Material

Supplementary Information

## Figures and Tables

**Table 1:** List of transcription factor binding element sequences.

Transcription factor	Sequence
NFκB	(GGGACTTTCC) × 8
SMAD2/3	(AGCCAGACA) × 8
SMAD1/5/8	(CGCGGCGCCAGCCTGACAGCCCG) × 6
HIF	(TACGTGCT) × 8
TCF/LEF	(AGATCAAAGGGGGTA) × 8
ER	(GTCAGGTCACAGTGACCTGAT) × 4
p53	(AGACATGTCCAGACATGTCCGAACATGTCCCAACATGTTGT) × 4
AP-1	(TGAGTCAG) × 8
FOXO1	(GATCAAGTAAACAACTATGTAAACAA) × 4
STAT3	(GTCGACATTTCCCGTAAATCGTCGA) × 4
GLI	(GACCACCCAC) × 8
NRF2	(TCACAGTGACTCAGCAAAATT) × 8
AhR	(TGAGTTCTCACGCTAGCAGAT) × 8
NFAT	(GGAGGAAAAACTGTTTCATACAGAAGGCGT) × 4
GR	(GGTACATTTTGTTCT) × 8
Notch	(CGTGGGAA) × 8
