# Supplementary material for: In vivo bioimaging with tissue-specific transcription factor activated luciferase reporters
Source: Sci Rep. 2015 Jul 3;5:11842. doi: 10.1038/srep11842 (PMC4490336; doi:10.1038/srep11842)
Supplement: Supplementary Information [file srep11842-s1.pdf]

In vivo bioimaging with tissue-specific transcription factor activated luciferase reporters

Suzanne MK Buckley[1], Juliette MKM Delhove[2,6], Dany P Perocheau[1], Rajvinder Karda[1], Ahad A Rahim[3], Steven J Howe[4], Natalie J Ward[1], Mark A Birrell[5], Maria G Belvisi[5], Patrick Arbuthnot[6], Mark R Johnson[7], Simon N Waddington[1,6], & Tristan R McKay[2]

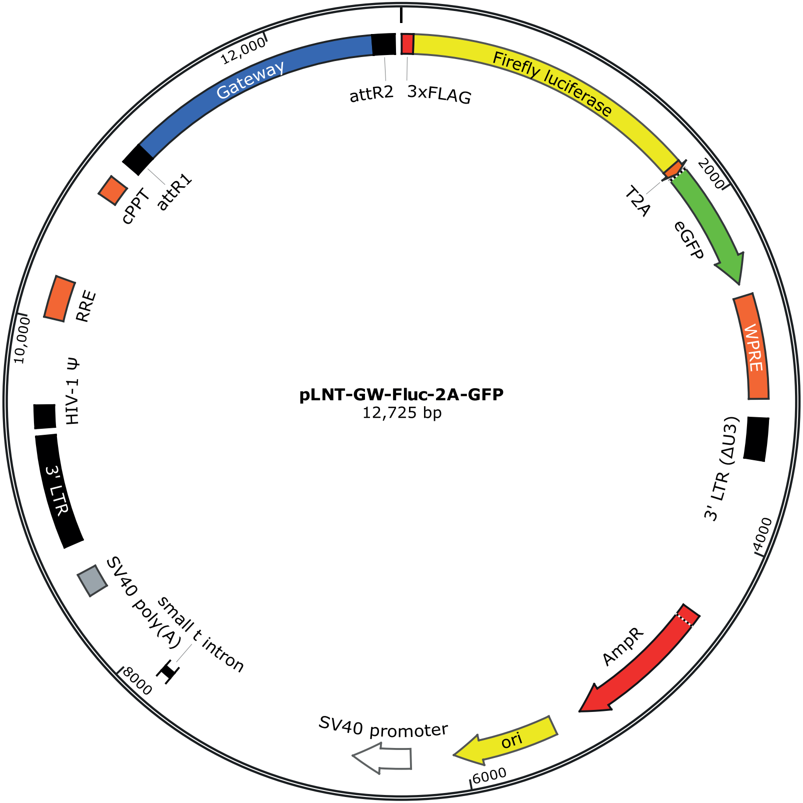

Supplementary Figure 1. Schematic representation of the lentiviral bicistronic reporter construct. The lentivirus vector is based on a second generation cassette incorporating a U3 deleted 3'-long terminal repeat (LTR). Packaged between the lentiviral LTRs is a Gateway cloning destination site upstream of a bicistronic in vivo optimized firefly luciferase-2A-eGFP reporter cassette including a FLAG tag. Gene expression is enhanced by the presence of an upstream central polypurine tract (cPPT) and a downstream woodchuck hepatitis virus post-transcriptional regulatory element (WPRE)

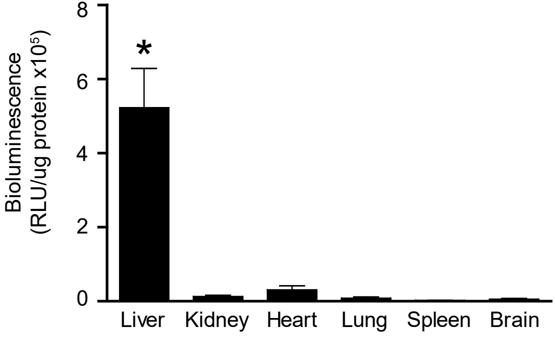

Supplementary Figure 2. Intravascular injection of VSV-G pseudotyped vector in neonatal mice results in liver targeted transduction. To examine vector biodistribution, neonatal mice received intravenous injection of VSV-G pseudotyped lentiviral vector (≈2×10^7 vp/mouse) containing luciferase under the control of the spleen focus forming virus (SFFV) constitutive promoter. At 80 days of age, mice underwent terminal exsanguination and PBS perfusion. Tissues were collected, and luminometry used to quantitate luciferase activity normalized to total protein. Detection of ex vivo luminescence was significantly higher in liver than in kidney, heart, lung, spleen or brain (*P<0.01, One-way ANOVA with Neumann-Keuls post-hoc test).

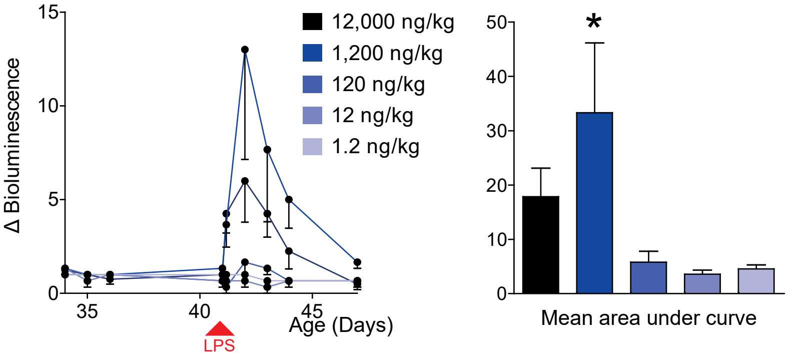

Supplementary Figure 3. LPS dose response. Outbred mice received neonatal intravenous injection of ≈2×10[7] vp/mouse of VSV-G pseudotyped vector to achieve liver-targeted expression of NFκB reporter. At 42 days of age, mice received a range of concentrations of i.p. LPS (1.2-12,000 ng/ml, n=3 per group except n=4 for 12,000 ng/ml). (a) The two highest LPS concentrations resulted in a strong increase in bioluminescent signal. Area under curve, calculated using the trapezoid rule, revelaed that BLI response was significantly higher at 1,200 ng/kg compared with all other doses (*P<0.05, One-way ANOVA with Neumann-Keuls post-hoc test).

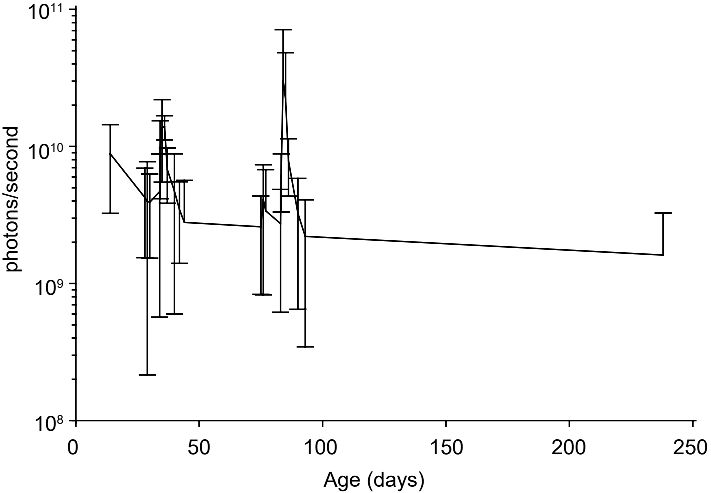

Supplementary Figure 4. Lipopolysaccharide (LPS)-mediated induction of NFκB-activated reporter cassette in targeted organs of reporter mice. This figure presents non-normalized data from which the graph in Figure 3ci was derived. Preparations of NFκB reporter lentiviral vector pseudotyped with VSV-G were injected intravenously into outbred mice (n=9). After establishing a baseline at 34 days, reporter mice were challenged with LPS and BLI measured continually thereafter. Baseline BLI was re-established in liver specific animals and were re-stimulated at 83 days and imaged over the same timeframe then once more at 8 months.

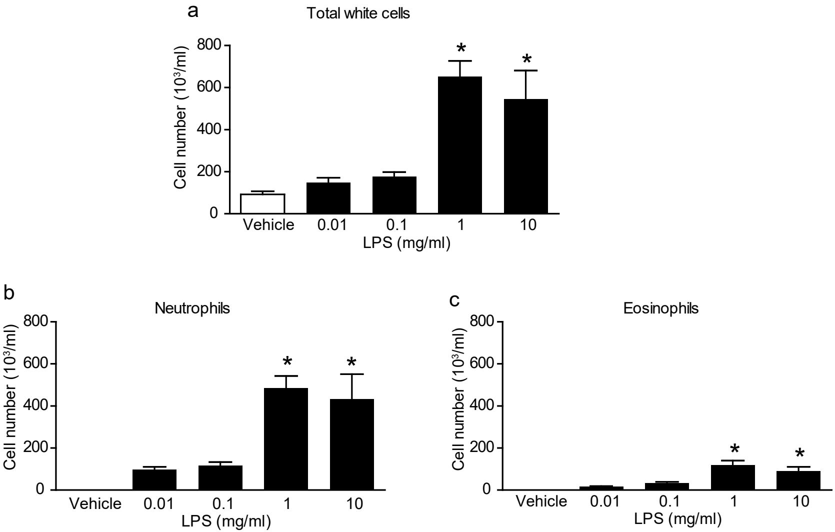

Supplementary Figure 5. Minimal efficacious dose of nebulized LPS for outbred mice. Male outbred mice (n=6 per group) were exposed to aerosolized vehicle or increasing doses of LPS for 30 minutes. Bronchoalveolar lavage fluid was collected at different time points after the exposure. The trachea was cannulated (Teflon precision dispensing tips, From Adhesive dispensers Ltd, UK) and then lavaged with 0.3 ml RPMI 1640 (Invitrogen, UK) three times, and the lavage fluid pooled for cell counting. Total white cell numbers in the BAL and lung tissue samples were determined on the Sysmex F820 hematology analyser. Cytospins of these samples were prepared by centrifugation of 100 µl aliquots in a cytospin (Shandon, Runcorn, UK) at 700 rpm for 5 min at low acceleration at room temperature. Slides were fixed and stained on a Hematek 2000 (Ames Co., Elkhart, IN) with modified Wright-Giemsa stain. Fourpart differential counts on 200 cells per slide were performed according to standard morphological criteria, and the percentage of eosinophils, lymphomononuclear cells, and neutrophils was determined (a) Total white cell, (b) neutrophil and (c) eosinophil counts are represented as mean ± SEM (*$P<0.05$ versus vehicle-challenged controls, One-way ANOVA with Neumann-Keuls post-hoc test)

Supplementary Table 1: Cloning PCR Primers

3xFLAG (F1)	CTGGGGCCACGAGGATCCGCCACCATGGACTACAAAGACCATGACGGTGATTATAAAGATC
3xFLAG (R1)	TTCTTGGCGTCCTCCATGCTGCCGCCGCTCTTG
FLuc (F2)	GCAGCATGGAGGACGCCAAGAACATCAAGAAGGG
FLuc (R2)	CTGCGCGGATCTTGCCGCCCTTCTTGGCC
2A-eGFP (F3)	AAGGGCGGCAAGATCCGCGCAGAGGGCCGGGGCTCAT
2A-eGFP (R3)	GTCAGCTGGGCAATGCATACTAGTTTGTTGAGTCAAAACTAGAGCCTGGACC
MP (F)	GATGCTCTCGAGGGGCTATAAAAGGGGGTGGGGGCGCGTTCGTCCTCACTCTCTTCC
MP (R)	GTCGAGCTCGAGGGAAGAGAGTGAGGACGAACGCGCCCCCACCCCCTTTTATAGCCC

Supplementary Table 2: Details of agonists & inhibitors

Agonist/Inhibitor	Supplier (Cat.)	Concentration	Activation Duration (hours)
LPS	InvivoGen (tlrl-3pelps)	100 ng/µl	4
Activin A	Peprotech (120-14)	100 ng/µl	72
BMP-2A	R&D Systems (355-BM)	100 ng/µl	72
$CoCl_2$	Sigma-Aldrich (C2644)	100 mM	12
LIF	R&D Systems (7734-LF)	10 µM	1
Nutlin-3A	Sigma-Aldrich (N6287)	25 µM	24
PMA	Sigma-Aldrich ((P1585)	10 ng/ml	24
LiCl	Sigma-Aldrich (203637)	50 mM	24
LY294002	Merck Millipore (440202)	20 mM	72
Purmorphamine	Stemgent (04-0009)	10 µM	4
Dexamethasone	Sigma-Aldrich (D4902)	100 nM	72
β-estradiol	Sigma-Aldrich (E2257)	100 nM	15
PMA & Ionomycin	Sigma-Aldrich/Tocris (2092)	10 ng/ml & 0.5 µM	24
TCDD	Sigma-Aldrich (48599)	10 nM	24
H_2O_2	Sigma-Aldrich (216763)	50 mM	0.3
